# Development and validation of multiple machine learning algorithms for differentiating primary central nervous system lymphoma from adult-type diffuse glioma: an interpretable and multicenter study

**DOI:** 10.3389/fonc.2025.1713099

**Published:** 2026-01-07

**Authors:** Yuanzi Liang, Junqi Hu, Tianhui Wu, Dong Bai, Zhiqun Wang

**Affiliations:** 1 Department of Radiology, Aerospace Center Hospital, Beijing, China; 2 Department of Nephrology, Aerospace Center Hospital, Beijing, China; 3 Department of Medical Imaging Center, Affiliated Hospital of Inner Mongolia Minzu University, Tongliao, China

**Keywords:** adult-type diffuse glioma, Interpretability, machine learning, MRI, primary central nervous system lymphoma

## Abstract

**Background:**

Preoperative differentiation of primary central nervous system lymphoma (PCNSL) from adult-type diffuse glioma(ADG) is important to guide neurosurgical decision-making.To develop and validate a MRI–based interpretable radiomic-clinical(Rad-Clinic) fusion model to differentiate PCNSL from ADG by seven machine learning algorithms.

**Methods:**

In this retrospective study, we recruited 165 patients who underwent preoperative conventional MRI(CET1WI, FLAIR, DWI, ADC) with PCNSL and ADG from two institutions (115 in the training cohort and 50 in the external validation cohort). we selected seven machine learning algorithms to construct a framework incorporating radiomic features and clinical parameters. SHapley Additive exPlanations (SHAP) values elucidated feature contributions, and a radiomic nomogram was developed for clinical translation.

**Results:**

The CET1WI+DWI+FLAIR fusion model exhibited optimal performance among all the single-sequence and multi-sequence radiomic models, and the AUC for external validation cohort were 0.871. But the Rad-Clinic fusion model performed well in differentiating PCNSL from ADG, and the AUC for the training and external validation cohort were 0.973 and 0.940, outperforming radiomic model and clinical model.SHAP summary plot illustrated the feature’s value affected the feature’simpact attributed to the Rad-Clinic fusion model.The nomogram demonstrated clinical interpretability through visualised risk stratification.

**Conclusion:**

An interpretable Rad-Clinic fusion model enables accurate preoperative to differentiate PCNSL from ADG, and may assist improve clinical decision-making.

## Introduction

Primary central nervous system lymphoma (PCNSL) and glioma represent the two most prevalent primary malignant intracranial neoplasms (1). The fifth edition of the the 2021 World Health Organization (WHO) Classification of Tumors of the Central Nervous System (CNS) introduced substantial revisions to the molecularly-defined classification of glioma subtypes, with adult-type diffuse glioma (ADG) emerging as the most common histological subtype (2, 3). PCNSL is predominantly managed through non-surgical therapeutic modalities, including chemotherapeutic regimens and targeted therapies such as high-dose methotrexate combined with consolidation chemotherapy and whole-brain radiotherapy (4). In contrast, the standard treatment paradigm for ADG involves maximal safe tumor resection followed by adjuvant radiotherapy and chemotherapy. Given these fundamentally divergent therapeutic approaches between the two malignancies, accurate preoperative differential diagnosis becomes imperative for optimal clinical decision-making and treatment stratification (5).

Although the characteristic magnetic resonance imaging(MRI) features of ADG and PCNSL have been extensively documented in the literature, the majority of clinical presentations manifest atypical radiological profiles, posing significant diagnostic challenges. This diagnostic ambiguity becomes particularly pronounced and clinically consequential when neoplasms arise in the central core regions of the cerebral hemispheres, where preoperative differentiation between these entities assumes heightened clinical significance (6). Consequently, accurate preoperative differentiation PCNSL from ADG is critical for guiding neurosurgical management strategies, avoiding unnecessary and potentially detrimental surgical interventions, and ultimately optimizing clinical outcomes, healthcare quality, and cost-effectiveness.

Radiomics extracts quantitative and reproducible features from medical images in high-throughput, complex modalities that are difficult to identify or quantify visually, which may be associated with a specific disease, and is used for tumor diagnosis, grading, efficacy assessment, and prognosis prediction by building predictive models (7–9). Currently, machine learning algorithms have been used with good success as a tool to differentiate PCNSL from ADG (10), and advanced sequences help to identify complex diseases (11), but these advanced examination sequences require additional costs and are not routinely used as sequences in clinical examinations of patients. Therefore, machine learning is applied on the basis of routine MRI sequences for a comprehensive analysis of tumors, which can reflect the cellular composition and heterogeneity of tumors by extracting radiomic features (12, 13). Constructing models by one or more machine learning algorithms for the characterization problem based on MRI routine sequences also includes the study and comparison of individual sequences or combined sequences, so the results of machine learning models developed based on MRI are not the identical, and the problem of differentiating PCNSL from ADG is still unresolved and un-recognized (14, 15). Therefore, further studies are required to determine the machine learning methods and the selection of the optimal sequence or combination of sequences for MRI. However, limited sample size and lack of interpretability of ml-based models limit the application of radiomic-based studies in clinical practice.The SHapley Additive exPlanations (SHAP) approach can help to solve this problem (16–18). Thus, the combination of SHAP and radiomics can over interpret the models.

This study aims to develop and validate multiple machine learning algorithms based on multiparametric MRI to discriminate PCNSL from ADG, and find the model with optimal performance by incorporating clinical factors and verified the generalizability of models by external validation.The SHAP algorithm is used to explore the interpretability of the models.

## Materials and methods

### Patients

**Figure 1:**
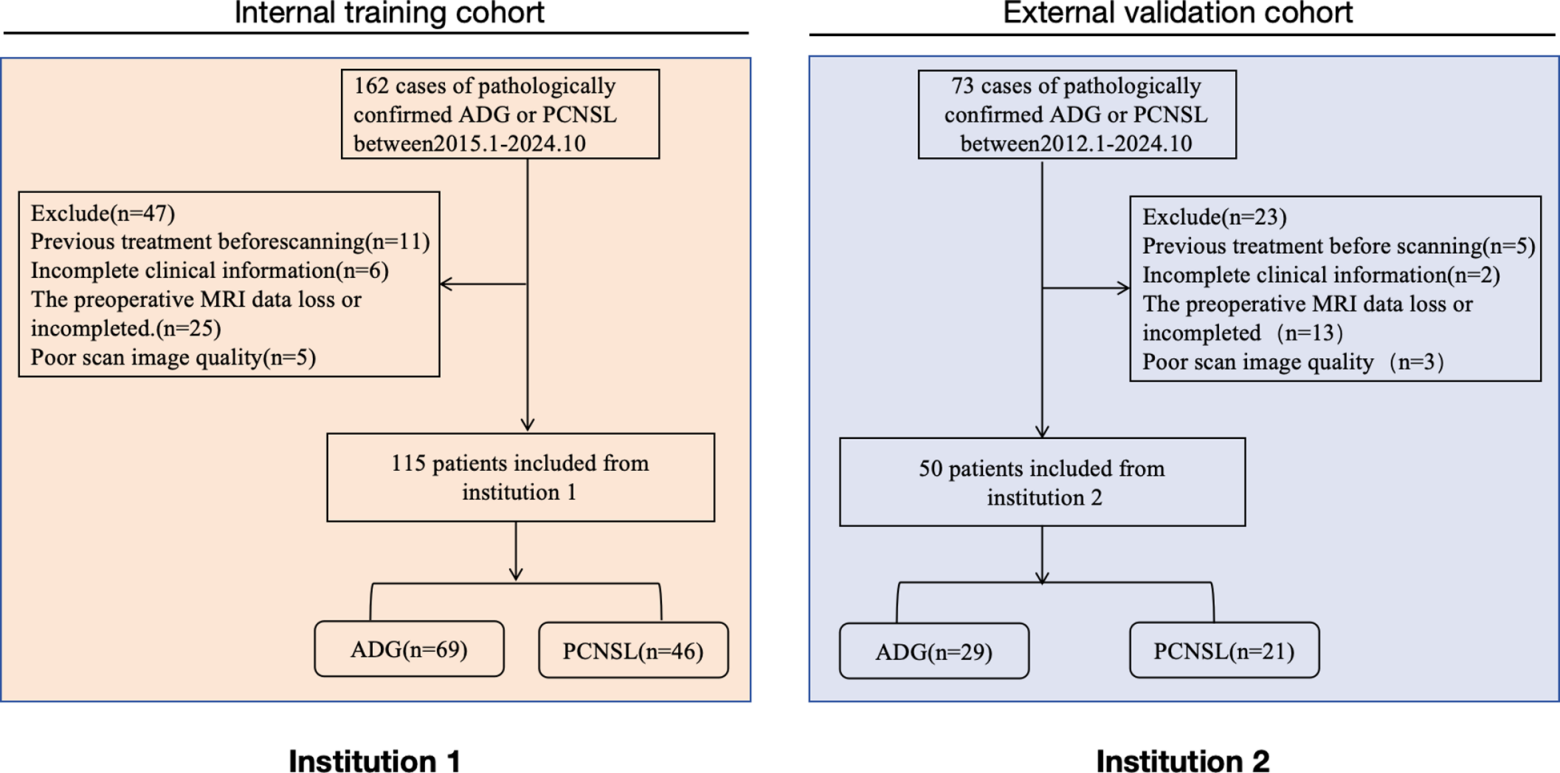
Recruitment pathway of the patient selection process from the two participating medical institutions of this study.

This retrospective multicenter study received ethical approval from the institutional review boards, and the informed consent was waived. We systematically reviewed all ADGs and PCNSL patients who underwent preoperative conventional MRI protocols whic including contrast-enhanced T1-weighted imaging(CET1WI), fluid attenuated inversion recovery(FLAIR), diffusion-weighted imaging(DWI) and apparent diffusion coefficient(ADC) between January 2015 and July 2024 across both institutions. ADG encompasses IDH-mutant astrocytoma, oligodendroglioma, and IDH-wildtype glioblastoma, for detailed information on the specific stratification of ADG, add details in **Supplementary Information 1**. The detailed inclusion and exclusion criteria are shown in **Figure 1**.We used Institution 1 as the training cohort and we used Institution 2 as the external validation cohort. Detailed MRI acquisition parameters across different scanner platforms are provided in **Supplementary Tables S1, S2**. A total of 165 patients were finally included in the two centers, with a total of 115 patients enrolled in Institution 1, including 69 patients with ADGs and 46 patients with PCNSL, and a total of 50 patients enrolled in Institution 2, including 29 patients with ADGs and 21 patients with PCNSL.Demographic and clinical characteristics were extracted from electronic medical records, including age, sex, and tumor morphological features (enhancement, number, location, edema and cystic).

### Data preprocessing and feature extraction

As the differences between centers and inter-scanner, we employed Combat during preprocessing to correct batch effects and achieve harmonization, yielding favorable results. To demonstrate this, we have included before and after PCA visualizations in the **Supplementary Figures S1A-D**. The images were resampled, and grayscale discretized prior to feature extraction. All images were resampled to 1 mm×1 mm×1 mm for the same resolution.To standardize the MR images of all sequences, the mean and standard deviation of the intensities in each MRI volume image were calculated and each value was standardized by the z-score method, which consists of subtracting the mean intensity and dividing by the standard deviation of the intensity (15).The images were analyzed separately and independently by two radiologists with 10 years of experience in neurological MRI diagnosis using a double-blind method.The region of interest (ROI) was manually segmented slice by slice on MRI images by using ITK-SNAP (http://www.itksnap.org);Areas of edema and necrosis were excluded so that only tumor was included.When outlining ROI for different sequences and simultaneously referring to image features of other sequences to ensure the accuracy of ROI.When outlining ROI on FLAIR images, the ROIs shall be adjusted by identifying the solid portion of the tumor against DWI and CET1WI images (19). Since the results of radiomic feature calculations depend on the ROI outlines depicted by radiologists, both test-retest analysis and inter-radiologist analysis should be applied to assess the robustness of all features. Based on 20 randomly selected patients, a test-retest analysis was performed in which each patient’s ROI was segmented twice by a radiologist. The dataset used for inter-evaluator analysis consisted of 20 patients selected as described above, where each patient’s ROI was split independently by two radiologists.

Radiomic features were calculated based on the specified ROIs (20). We used PyRadiomics package (version 3.0) for Python (version 3.7.3) for radiomic feature extraction, and we extracted radiomic features from CET1WI, FLAIR, DWI, and ADC of tumor lesions. The first-order statistical features describe the distribution of voxel intensities within the tumor, and the texture features use a grayscale matrix to represent the spatial heterogeneity of the tumor.

### Feature selection and model construction

Feature selection was needed to reduce overfitting, redundancy, or any other type of bias. Radiomic features were calculated after each depiction and intra- and inter-observer reproducibility was determined for each feature. Features with low reproducibility (interclass correlation coefficient (ICC) below 0.75 were excluded) (21–23). All eigenvalues were normalized using the z-score transformation to reduce potential differences in eigenvalues between the two cohorts (24). Spearman correlation coefficient was applied to calculate the correlation between features and one of the features with a correlation coefficient greater than 0.9 between any two features was retained (25). The least absolute shrinkage and selection operator (LASSO) algorithm is further applied to filter the best radiomic features by a ten-fold cross-validation method (15). Finally, we selected seven machine learning classifiers to construct the radiomic model, including Logistic Regression (LR), Support Vector Machine (SVM), Random Forest (RF), K-Nearest Neighbors (KNN), Extra Trees, LightGBM and Multilayer Perceptron (MLP).The hyperparameters for each classifier are provided in **Supplementary Information 2**. To improve the performance of the radiomic model, clinical baseline features were selected to construct the clinical-based model. Univariate and multivariate analyses were performed to identify independent risk factors for differentiating PCNSL from ADG, and finally, clinical indicators were integrated into the radiomic model to construct a Rad-Clinic fusion model.

### Explanation and visualization of rad-clinic model

The SHAP method enables the results of the model to be understood in an interpretable manner, providing visually concise graphs by representing the range and distribution of the importance of features to the model outputs and by correlating the values of features with the impact of features, thus enhancing model transparency by providing global and local interpretability (22, 23). The features are ranked in order of importance, with higher ranked features contributing more to the model. Each point representing the SHAP value of each feature for a patient is plotted horizontally and stacked vertically to show the density of the same SHAP value (25). The method elucidates the most influential variables, thereby significantly improving the interpretability of the model (26). Enhanced understanding of key predictors allows for informed assessment of their contribution to predictive outcomes in the training cohort.

### Construction of a fusion model-based nomogram

To visualise the classification assessment, we conducted a logistic regression analysis to build a nomogram based on the Rad-Clinic fusion model and demographic characteristics.

### Statistical analysis

Python (version 3.7.3) and SPSS (v22.0, IBM) statistical packages were used for statistical analysis. We used independent samples t-tests and Mann-Whitney tests to analyze variables between the training cohort and the external validation cohort, and univariate and multivariate logistic regression to analyze continuous and categorical variables between different groups of patients. Categorical variables were compared using the chi-square test or Fisher’s exact test. Univariate and multivariate analyses were performed for various factors. The area under the curve (AUC) of the subjects was used to assess the discriminatory power of the model. Accuracy, sensitivity (SEN) and specificity (SPE) were calculated to quantify the discriminative power of the predictive models. Delong test was used to compare AUC values between models, and Net Reclassification Improvement (NRI) and Integrated Discrimination Improvement (IDI) were calculated to quantify the improvement in discrimination performance. To assess how well the predictions of the models match the actual results, calibration curve analysis was performed and quantified with the Hosmer-Lemeshow(HL) test. In addition, decision curve analysis (DCA) was used to quantify the net benefit at different threshold probabilities, thus highlighting the clinical applicability of the model. The SHAP method was used to explain the influence of features on the predictive model, elucidating the most influential variables, thus significantly improving the interpretability of the model.

## Results

### Patient characteristics

**Table 1** summarizes the baseline characteristics of all participants.In the training cohort, significant differences were observed in age, tumor intensification pattern, number of tumors, tumor location, and tumor cystic necrosis (p<0.05) between the groups with ADG and PCNSL. In the external validation cohort, age, tumor intensification pattern, and tumor cystic necrosis demonstrated statistically significant differences (p<0.05). Comprehensive comparisons of baseline characteristics between the internal training and external validation cohort revealed no significant differences between the two cohorts. Detailed results of the stratified analysis for GBM and PCNSL are provided in the **Supplementary Table S3**.

**Table 1:** Participants characteristics.

Variables	Training cohort			External validation cohort		
	ADGs (n=69)	PCNSL (n=46)	P value	ADGs (n=29)	PCNSL (n=21)	P value
Age	48.39 ± 15.92	61.17 ± 13.90	<0.001	46.17 ± 16.18	58.14 ± 10.69	0.006
Gender			0.373			1.0
Female	31(44.93)	16(34.78)		13(44.83)	9(42.86)	
Male	38(55.07)	30(65.22)		16(55.17)	12(57.14)	
Enhancement			<0.001			<0.001
Nonenhancing	11(15.94)	4(8.70)		7(24.41)	0	
Ringlike enhancing	44(63.77)	5(10.87)		20(68.97)	1(4.76)	
Solidlike enhancing	14(20.29)	37(80.43)		2(6.90)	20(95.24)	
Number			0.008			0.137
Single	51(73.91)	22(47.83)		21(72.41)	10(47.62)	
Multiple	18(26.09)	24(52.17)		8(27.59)	11(52.38)	
Localization			0.03			0.04
Midline	31(44.93)	31(67.39)		16(55.17)	14(66.67)	
Peripheral	38(55.07)	15(32.61)		13(44.83)	7(33.33)	
edema(<1.5)			1.0			0.497
Yes	16(23.19)	10(21.74)		6(20.69)	7(33.33)	
No	53(76.81)	36(78.26)		23(79.31)	14(66.67)	
Cystic			0.003			0.005
Present	23(33.33)	29(63.04)		8(27.59)	15(71.43)	
Absent	46(66.67)	17(36.96)		21(72.41)	6(28.57)	

### Feature selection

**Figure 2:**
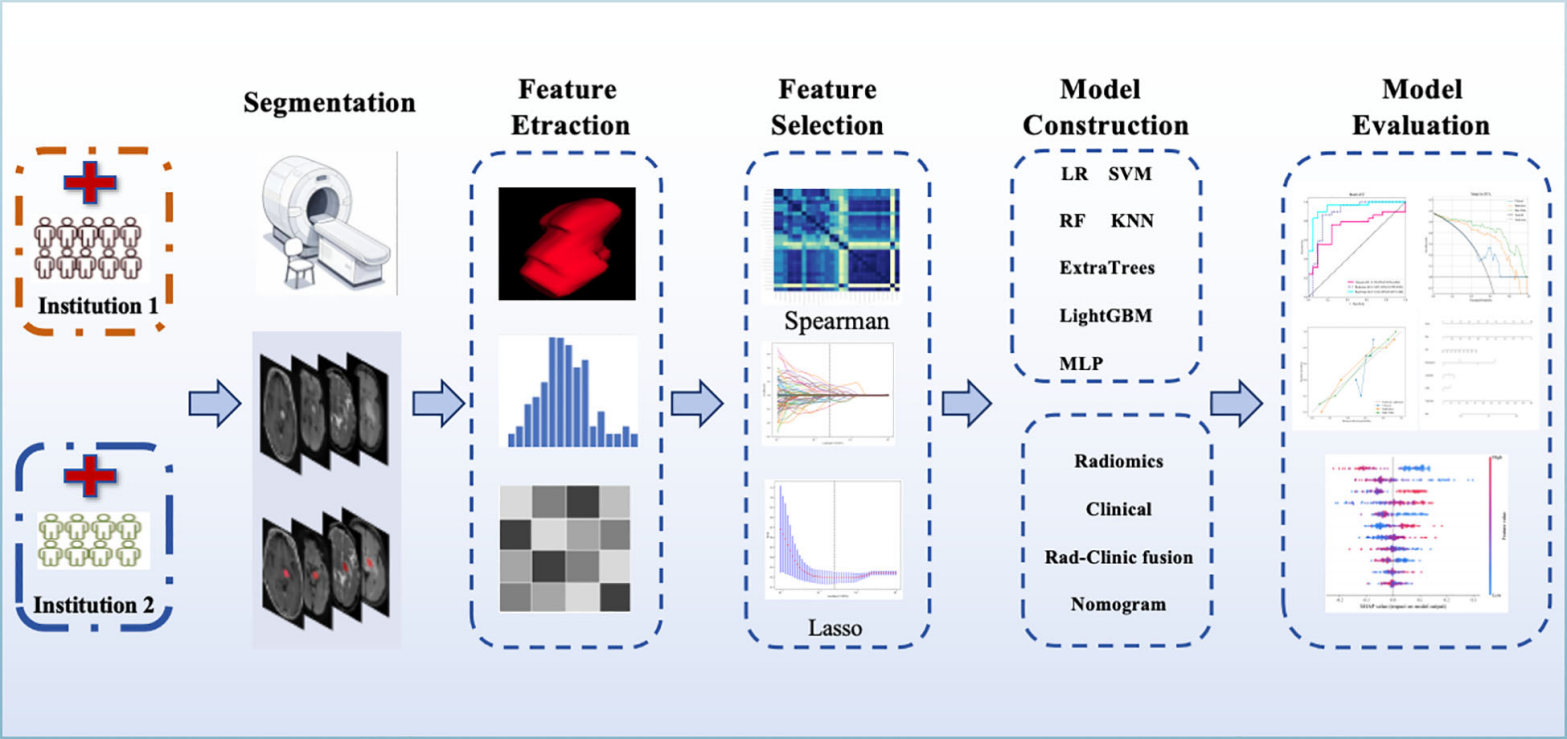
The workflow of radiomic model construction process.

The workflow for constructing the radiomic model is shown in **Figure 2**. Radiomic features were extracted from tumor lesions across four MRI sequences(CET1WI, FLAIR, DWI, and ADC). This enabled the development of four single-sequence radiomic models and eleven multi-sequence radiomic models. The multi-sequence models were created through multivariate logistic regression, integrating all possible combinations of the single-sequence models. They are composed of the linearly weighted sum of outputs from the single-sequence models.

Individual features retained within each of the CET1WI, FLAIR, DWI, and ADC sequences are detailed in **Supplementary Table S4** for single-sequence and multi-sequence radiomic models.

### Performance of radiomic−based machine learning model

In these machine learning algorithms, the combined CET1WI+DWI+FLAIR model using the SVM method demonstrated superior diagnostic efficiency, achieving AUC values of 0.975 for the training set and 0.871 for the validation set. The performance of other sequence models is detailed in the **Supplementary Table S5**.

**Figure 3:**
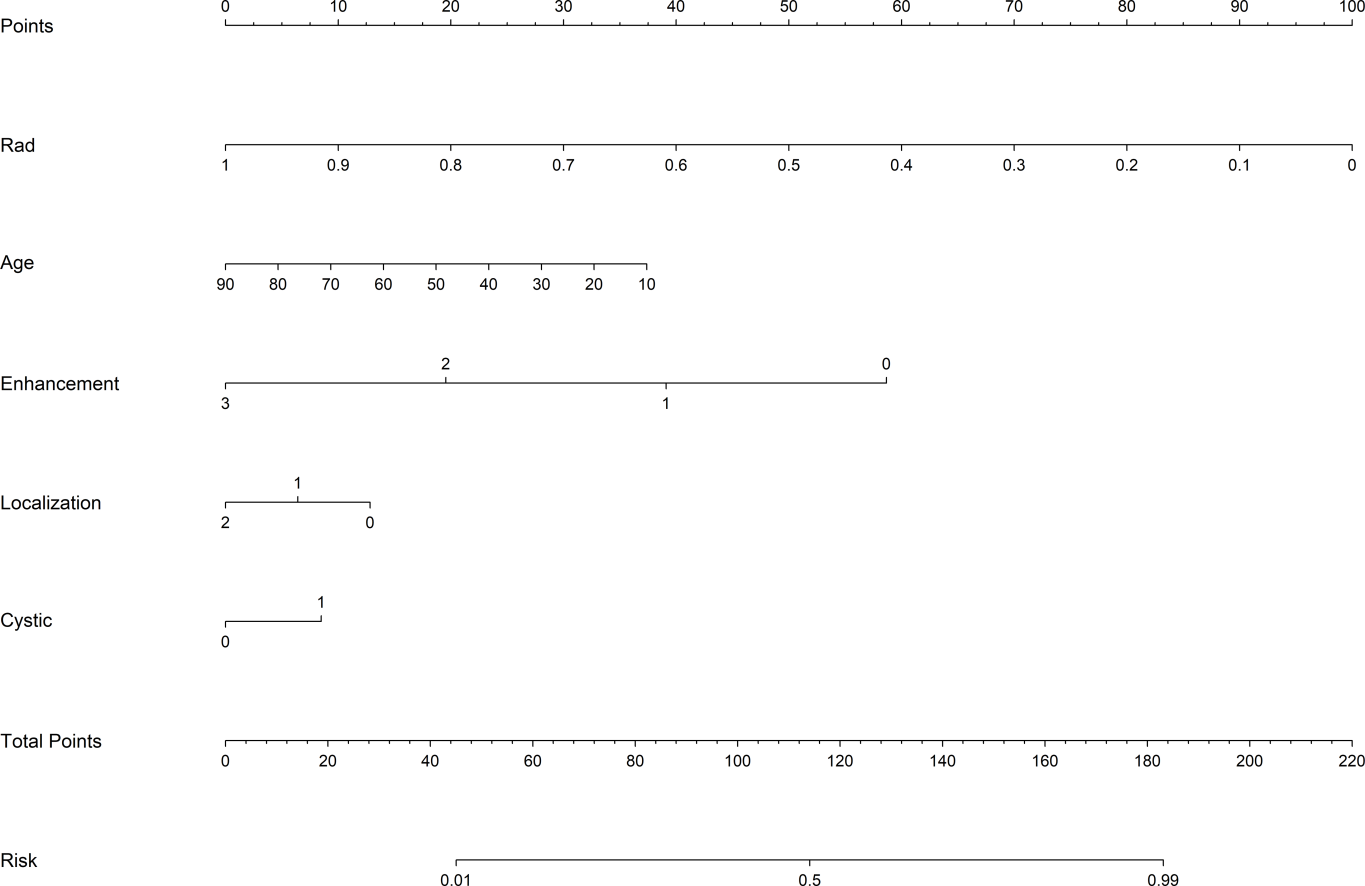
Nomogram of the Rad-Clinic fusion model for differentiating PCNSL from ADG.

**Figure 4:**
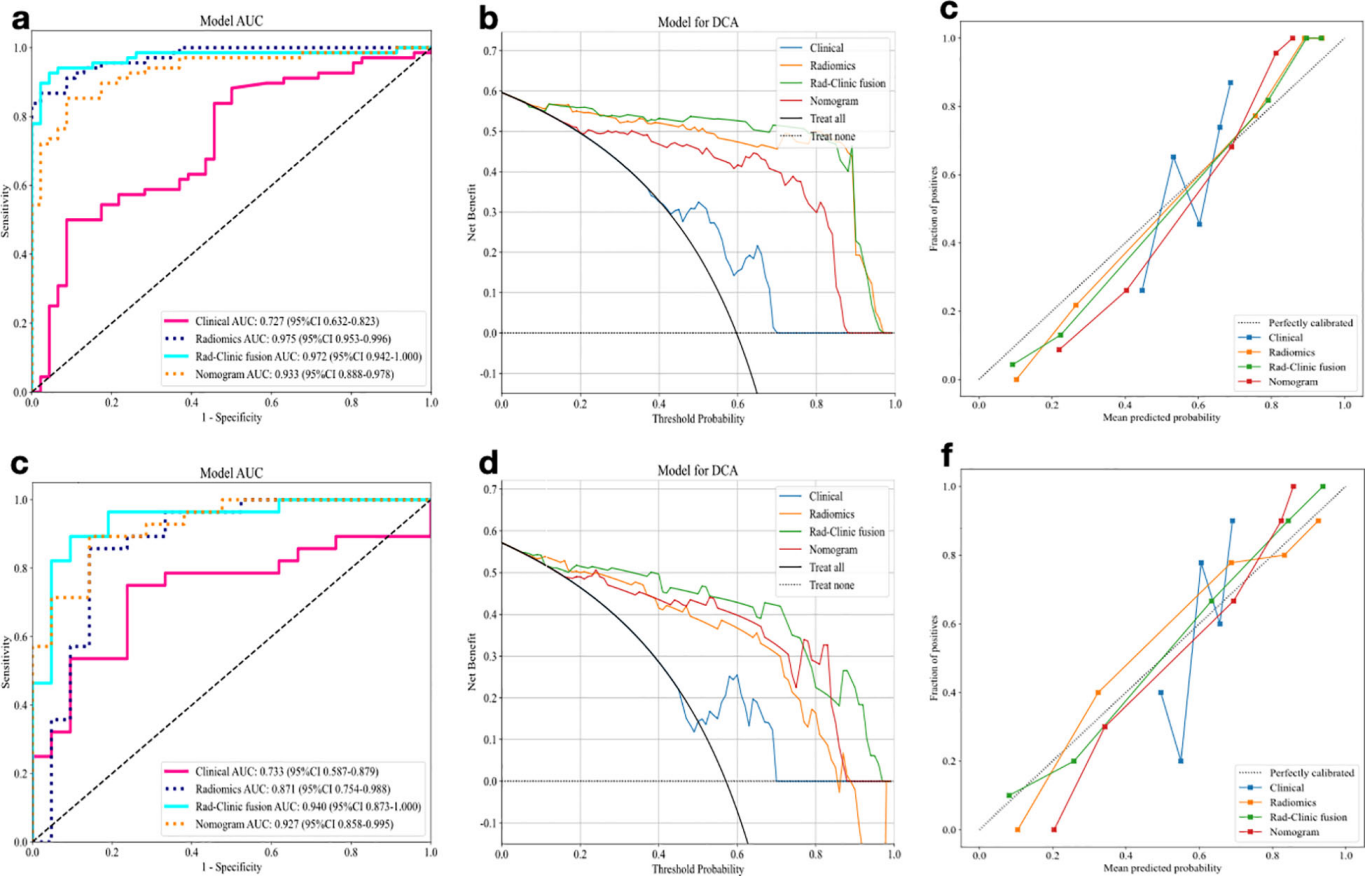
Comparison of the model’s performance in the training and validation cohort. **(A–C)** ROC curves, decision curve analysis and calibration curve of Radiomic model, Clinical model, Rad-Clinic fusion model and nomogram in training cohorts. **(D–F)** ROC curves, decision curve analysis and calibration curve of Radiomic model, Clinical model, Rad-Clinic fusion model and nomogram in validation cohorts.

Indicators with significant correlation with identifying ADG and PCNSL (age, mode of intensification, and tumor cystic changes) were used for the construction of the clinical model, and AUC values of 0.727 and 0.733 were obtained for the training and validation cohort.The clinical indicators were integrated into the radiomic model for the construction of the Rad-Clinic fusion model, and AUC values of 0.973 and 0.940 were obtained for the training and validation cohort. A nomogram (**Figure 3**) was created based on the independent clinical predictors and the best fusion model, and the AUC values of the nomogram training and validation cohort were 0.933 and 0.927, respectively.Therefore, the Rad-Clinic fusion model performed the best among all models. DeLong test, NRI and IDI were used to compare the diagnostic effectiveness of the models, and in the validation cohort results, the Delong test was statistically significant (p<0.05) for the radiomic model, fusion model and nomogram compared to the clinical model, but the Delong test was not statistically significant for the Rad-Clinic fusion model compared to the radiomic model.This may also be due to the insensitivity of the DeLong test to small samples, the NRI and IDI analyses further confirmed improved diagnostic efficiency with the Rad-Clinic fusion model.The Hosmer-Lemeshow goodness-of-fit test indicated a superior fit for the fusion model, corroborated by calibration curves, suggesting enhanced predictive accuracy. This improvement augments the clinical model, radiomic model, and overall predictive performance. The ROC curves, decision curve analyses (DCA), and calibration curves for the fusion model and nomogram are shown in **Figure 4**. The diagnostic performance details are summarized in **Table 2**.

**Table 2:** Performance of radiomic model, clinical model and rad-clinic fusion model.

Data set	Model	AUC	ACC	Sen	Spe	PPV	NPV
Training cohort	Clinical	0.727(0.632,0.823)	0.658(0.623, 0.714)	0.485(0.441, 0.606)	0.913(0.845,0.965)	0.892(0.820,0.947)	0.545(0.474,0.607)
	Radiomic	0.975(0.953, 0.996)	0.904(0.872, 0.943)	0.853(0.779, 0.901)	0.978(0.951,1.000)	0.983(0.961,1.000)	0.818(0.768,0.857)
	Rad-Clinic fusion	0.972(0.942,1.000)	0.930(0.891, 0.956)	0.912(0.796, 0.964)	0.957(0.936,0.994)	0.969(0.935,1.000)	0.880(0.816,0.932)
	Nomogram	0.933(0.888,0.978)	0.868(0.763, 0.899)	0.838(0.815, 0.890)	0.913(0.875,0.955)	0.934(0.888,0.992)	0.792(0.740,0.833)
Validation cohort	Clinical	0.733(0.587,0.879)	0.735(0.654, 0.792)	0.714(0.584, 0.765)	0.782(0.696,0.833)	0.800(0.765,0.823)	0.667(0.579,0.701)
	Radiomic	0.871(0.754,0.988)	0.837(0.787, 0.859)	0.821(0.783, 0.866)	0.857(0.835,0.912)	0.885(0.854,0.963)	0.783(0.762,0.818)
	Rad-Clinic fusion	0.940(0.873,1.000)	0.878(0.823, 0.936)	0.857(0.828, 0.912)	0.905(0.863,0.935)	0.923(0.878,0.962)	0.826(0.804,0.887)
	Nomogram	0.927(0.858,0.995)	0.857(0.804, 0.925)	0.857(0.834, 0.872)	0.857(0.817,0.906)	0.889(0.815,0.936)	0.818(0.780,0.832)

### Explanation and visualization of rad-clinic model

**Figure 5:**
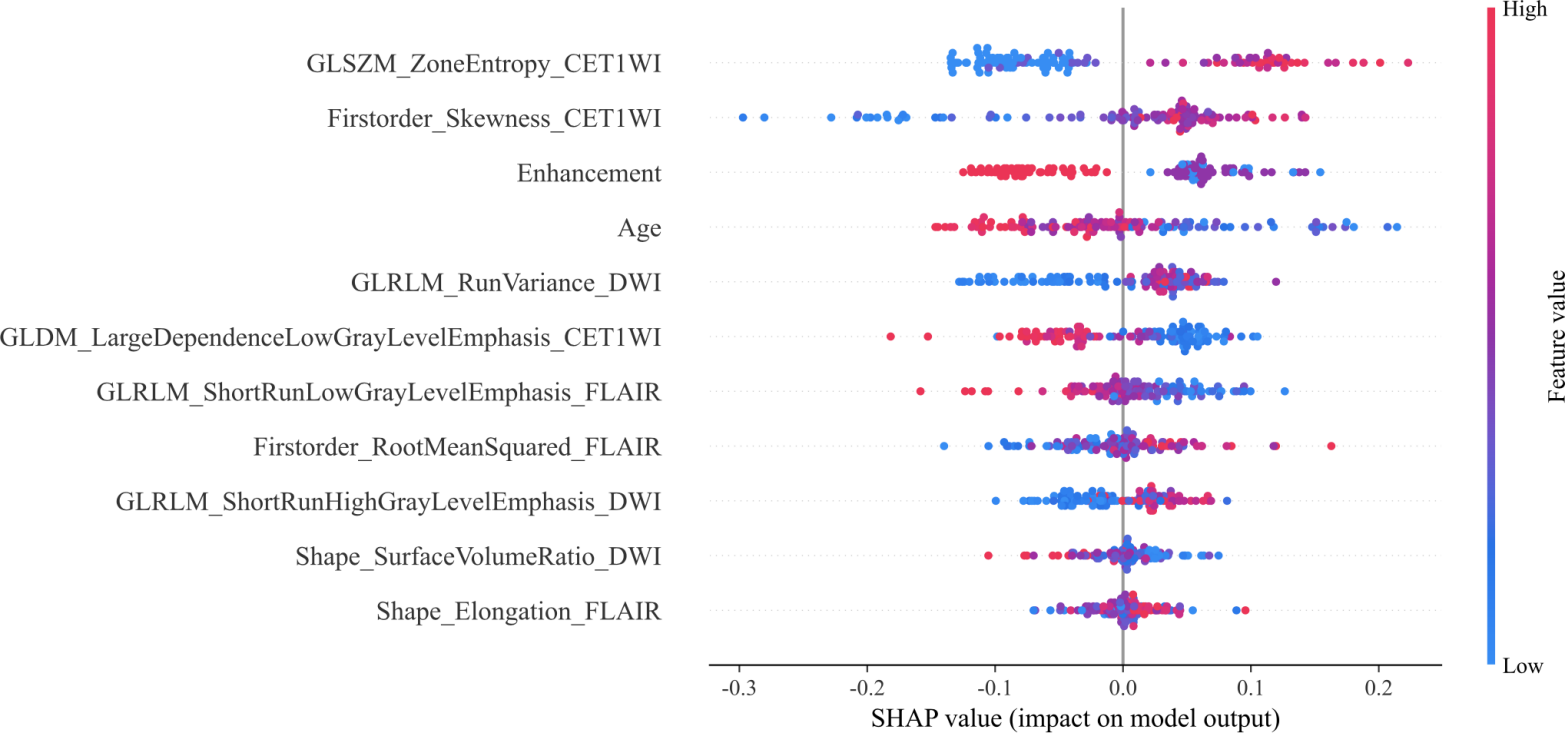
SHAP summary plot of Rad-Clinic fusion model.The plot illustrates the feature relevance and combined feature attributions to the models’ predictive performance.

We constructed the optimal Rad-Clinic model and used the SHAP method for visualizing and analyzing the differentiation between ADG and PCNSL. The feature ‘GLSZM_ZoneEntropy_CET1WI’ emerged as the most significant in distinguishing between these two conditions. Among clinical features, reinforcement modality and age also played vital roles. The statistical significance of ‘GLSZM_ZoneEntropy_CET1WI’ in differentiating ADG from PCNSL is shown in **Figure 5**, where the SHAP summary plot elucidates the cumulative impact of each variable.

**Figure 6:**
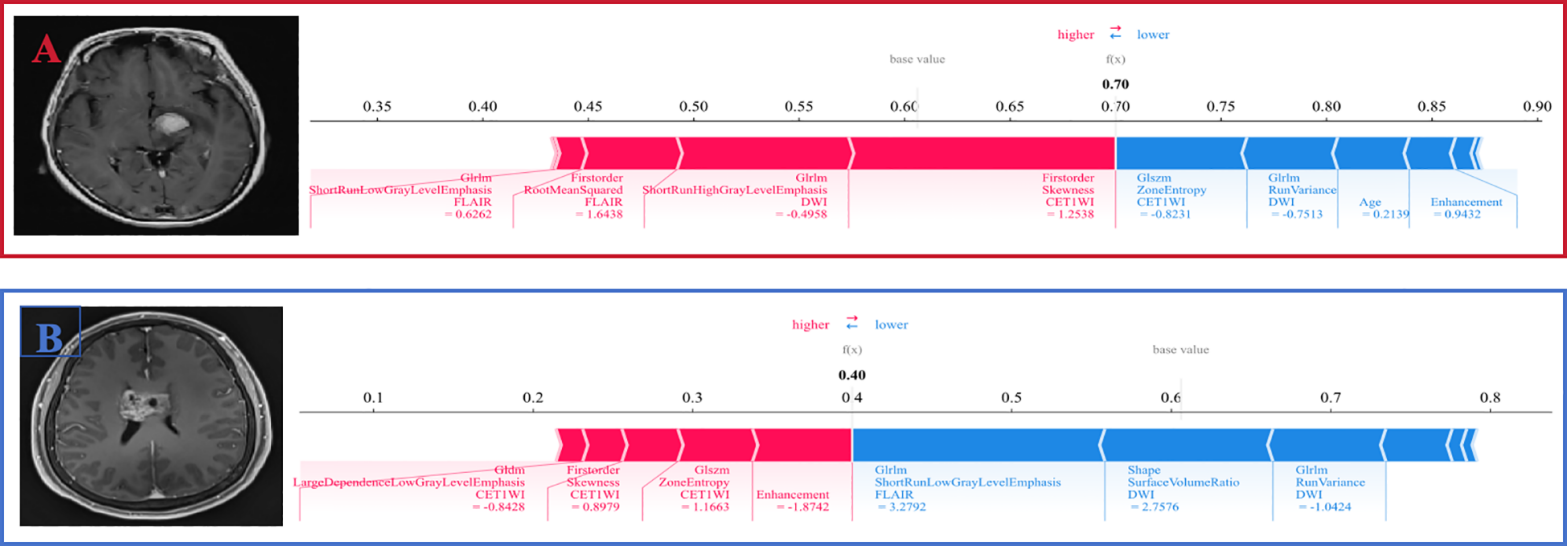
SHAP force plots explained how the Rad-Clinic fusion model differentiating PCNSL from ADG. The patient **(A)** was PCNSL, and the patient **(B)** was ADG. For instance, low feature value of GLSZM_ZoneEntropy_CET1WI contributed to the increase in the assessment probability of PCNSL. Patient **(B)** had a GLSZM_ZoneEntropy_CET1WI value of 1.1663, while a lower GLSZM_ZoneEntropy_CET1WI value of patient A (-0.8231).

**Figure 6** shows that in the evaluation of Patient A, the SHAP value was higher than the baseline value, indicating that this patient was an individual with lymphoma, and the characteristic arrow contributed to the quantitative assessment of PCNSL. The GLSZM_ZoneEntropy_CET1WI feature was negatively correlated with the SHAP value(-0.8231). Patient B was an individual with ADG, whose SHAP value was significantly lower than the baseline value, and the GLSZM_ZoneEntropy_CET1WI feature of this patient was positively correlated with the SHAP value(1.1663).

## Discussion

In this study, we developed a multi-parametric MRI-based machine learning Algorithm that integrates both radiomics and clinical data to preoperatively differentiating PCNSL from ADG. The combined Rad-Clinic model demonstrated superior diagnostic efficiency compared to standalone radiomic or clinical models, supported by external validation. Visualization techniques were applied to effectively present the predictive factors.

Seven machine learning classifiers(LR, SVM, RF, KNN, ExtraTrees, LightGBM and MLP) were utilized to construct an interpretable radiomic model differentiating PCNSL from ADG. Radiomic features were extracted from conventional MRI sequences, including CET1WI, FLAIR, DWI, and ADC. In the validation cohort, the combination model of CET1WI+DWI+FLAIR using SVM method the best diagnostic efficiency, with AUCs of 0.975 for training and 0.871 for external validation.Additionally, a clinical model was developed by integrating clinically significant indicators, achieving AUCs of 0.730 for the training and 0.735 for the validation cohort. These clinical indicators were incorporated into the radiomic model to construct the Rad-Clinic fusion model, resulting in AUCs of 0.972 for training and 0.940 for validation. A nomogram, based on independent clinical predictors and the optimal fusion model, provided AUCs of 0.933 and 0.927 for the training and validation cohort.The Rad-Clinic fusion model delivered the highest diagnostic performance among all models, significantly enhancing diagnostic efficiency. DCA and calibration curves further confirmed that the fusion model offered considerable benefits in differentiating ADG from PCNSL.

In this study, the SVM-based CET1WI model demonstrated the highest diagnostic efficiency among single-sequence models, and the combination of CET1WI+DWI+FLAIR based on the SVM method had the best diagnostic efficiency mong multi-sequence models. Notably, the feature ‘GLSZM_ZoneEntropy_CET1WI’ from the CET1WI sequence was the most significant contributor to the diagnostic model. Enhancement modality, which is inherently related to the CET1WI sequence, was statistically significant for diagnosis, underscoring the sequence’s critical role in differentiating PCNSL from ADG. Each MRI sequence capitalizes on the distinct biophysical characteristics of brain tumors. The CET1WI sequence, in particular, effectively highlights areas of enhancement and necrosis, reflecting the degree of blood-brain barrier disruption and contrast agent aggregation, thereby offering robust diagnostic potential for distinguishing PCNSL and ADG (27). Nevertheless, the higher diagnostic efficiency achieved by the CET1WI+DWI+FLAIR combination suggests the necessity of incorporating multiple MR sequences for optimal diagnostic performance, despite CET1WI is important for tumor diagnosis (28).Among baseline clinical features, age, enhancement, and tumor cystic changes were significantly associated with differential tumor diagnosis in both the training and validation cohorts. Age emerged as the most influential clinical feature, both in the nomogram and SHAP analysis, and is easily obtainable preoperatively, making it a common inclusion in joint models (29, 30). Tumor enhancement and cystic characteristics, as baseline imaging features, are derived from preoperative imaging. Typically, ADG presents with ring-shaped enhancement and frequent cystic necrosis, while PCNSL is characterized by solid enhancement and relatively rare cystic changes (31). This difference may relate to the pathophysiological mechanisms that ADG rapid growth can lead to ischemia and hypoxia-induced necrosis, completely destroying the blood-brain barrier in the necrotic area and resulting in non-enhancement at the center (1, 3, 32, 33). Around the periphery, viable tumor cells exhibit active proliferation and neovascularization, allowing contrast agents to leak in areas of increased vascular permeability, thus creating ring-shaped enhancement (34).

This study employed seven machine learning methods, with the SVM-based models demonstrating the highest diagnostic efficiency for both single-sequence CET1WI and multi-sequence fusion models of CET1WI+FLAIR+DWI. Despite these satisfactory results, visual demonstration was initially lacking. Therefore, we utilized SHAP analysis to elucidate how each feature influences model predictions, by visualizing the contribution value of each feature.SHAP values quantify the contribution of each feature to the model’s predictions, with positive values suggesting an increased likelihood of the predicted outcome and negative values a decreased likelihood (35). This study aims to address challenges of model interpretability and explainability. Interpretability refers to how well humans can understand or intuitively grasp a model’s output, while explainability pertains to the underlying mechanisms and logic of a machine learning system (26).We used SHAP to identify the two most influential features within the CET1WI sequence, including GLSZM and First-order features. GLSZM highlights homogeneous regions and tumor heterogeneity on a regional scale, reflecting the complexity and variation within the tumor, potentially linked to tumor aggressiveness or the immune microenvironment (36). First-order features, derived from grayscale values of tumor images, encompass various first-order statistics reflecting intensity distribution within the tumor and its internal heterogeneity.Furthermore, localized interpretation of individual patient assessments can be accomplished using SHAP force diagrams, which are faster and easier to use than nomograms (23). Clinicians can directly compare an individual patient’s SHAP values to baseline values. If an output SHAP value exceeds the baseline, a clinician may classify the patient as having PCNSL. SHAP force diagrams also visually demonstrate how features influence a patient’s assessment (25).Arrow color indicates the impact (red increases the likelihood of nonresponse), and arrow length signifies the feature’s contribution magnitude. These tools facilitate a nuanced understanding of feature roles in patient-specific assessments (23–26).

Previous research aimed at differentiating PCNSL from ADG has employed various machine learning models based on single-sequence MRI data, yielding inconsistent results (18). The emergence of multi-parametric MRI studies confirmed that models combining multiple sequences exhibit higher diagnostic efficiency than those relying on single-sequence data (15). For instance, one such study utilized a machine learning model incorporating multiple MRI sequences, achieving optimal diagnostic performance with a CET1WI+ADC combined model, displaying AUC values of 0.943 for the training and 0.935 for the validation cohort (19). Another study using cross-validation across multiple classifiers reported the highest diagnostic efficiency with an ADC+FLAIR+CET1WI model, achieving an AUC of 0.977 (37).While previous scholars have developed models using single or multiple machine learning methods to identify PCNSL and ADG through MRI data, these models often overlooked the integration and validation of clinical data alongside radiomic features (15, 19, 37). They lacked the ability to differentiate and assess the significance of clinical information in conjunction with imaging features for diagnosis. The incorporation of such features, alongside visualizing the model, enhances model completeness and provides more practical diagnostic guidance for clinicians.Our study addresses these gaps by using seven machine learning methods to develop and validate models using multi-parametric MRI sequences. We integrated clinical and imaging data to ensure comprehensive model development. Subsequently, we visualized the model using SHAP analysis, allowing for a nuanced understanding of feature influence, thus advancing the model’s applicability in clinical settings. Compared to previous studies, our approach provides a more holistic tool for clinicians seeking to differentiate PCNSL from ADG.

Despite the valuable insights gained from this study, several limitations remain. First, although data were sourced from two centers, the patient cohort size was relatively limited. Future research should focus on developing predictive models based on larger sample sizes to enhance robustness and generalizability. Second, as a retrospective study, this research may be subject to selection bias. To address this, future studies should incorporate data from multiple centers to facilitate prospective analyses, thereby improving the reliability and validity of the model.Third, the study predominantly relied on machine learning models, necessitating manual delineation of ROIs, which is time-consuming. Future efforts should prioritize the development of automatic or semi-automatic tumor segmentation techniques to streamline and expedite the process, enhancing efficiency and practicality in clinical settings.Finally, this study employed performing model selection and tuning within a single 10-fold cross-validation framework (without nesting), which may lead to slightly optimistic performance estimates. In future research, we will strive to adopt nested cross-validation to provide more rigorous work.

This study successfully developed and validated multiple machine learning models based on multiparametric MRI sequences, integrating radiomics and clinical indicators to construct interpretable models that can provide a reliable and noninvasive tool for preoperative differentiation of PCNSL and ADG.

## Data Availability

The raw data supporting the conclusions of this article will be made available by the authors, without undue reservation.
