# Supplementary material for: Development and validation of multiple machine learning algorithms for differentiating primary central nervous system lymphoma from adult-type diffuse glioma: an interpretable and multicenter study
Source: Front Oncol. 2026 Jan 7;15:1713099. doi: 10.3389/fonc.2025.1713099 (PMC12819316; doi:10.3389/fonc.2025.1713099)
Supplement: Supplementary file 1 [file DataSheet1.docx]

|  | TR(ms) | TE(ms) | FOV(mm^2^) | Matrix | Layer Thickness(mm) | Flip Angle |
| --- | --- | --- | --- | --- | --- | --- |
| CET1WI | 1800 | 9 | 240×240 | 320×320 | 5 | 150 |
| FLAIR | 4500 | 85 | 240×240 | 256×256 | 5 | 150 |
| DWI | 4300 | 64 | 240×240 | 164×164 | 5 | 180 |

**Table S1 MR scanning parameters for patients from institution 1**

Images were acquired by using 3.0-T MRI (skyra, Simens, Germany)

TR=Repetition time；TE=Echo time；FOV=Field of view；ADC maps were performed on the the workstation was generated automatically by DWI.

**Table S2 MR scanning parameters for patients from institution 2**

|  | TR(ms) | TE(ms) | FOV(mm^2^) | Matrix | Layer Thickness(mm) | Flip Angle |
| --- | --- | --- | --- | --- | --- | --- |
| CET1WI | 2050 | 8 | 240×240 | 256×256 | 5 | 150 |
| FLAIR | 8000 | 92 | 240×240 | 256×256 | 5 | 150 |
| DWI | 3400 | 75 | 240×240 | 192×192 | 5 | 180 |

Images were acquired by using 1.5-T MRI (Avanto, Simens, Germany)

TR=Repetition time；TE=Echo time；FOV=Field of view；ADC maps were performed on the the workstation was generated automatically by DWI.

**Table S3 Participants characteristics (GBM vs PCNSL)**

| Variable | Training cohort |  |  | External validation cohort | |  |
| --- | --- | --- | --- | --- | --- | --- |
|  | GBM(n=30) | PCNSL(n=46) | p value | GBM(n=12) | PCNSL(n=21) | p value |
| Age | 51.67±17.68 | 61.17±13.90 | 0.018 | 55.17±11.83 | 58.14±10.69 | 0.369 |
| Gender |  |  | 1.0 |  |  | 1.0 |
| Female | 11(36.67） | 16(34.78） |  | 5(41.67） | 9(42.86） |  |
| Male | 19(63.33） | 30(65.22） |  | 7(58.33） | 12(57.14） |  |
| Enhancement |  |  | <0.001 |  |  | <0.001 |
| Nonenhancing | 1(3.33） | 4(8.70） |  | 0 | 0 |  |
| Ringlike enhancing | 27(90.00） | 5(10.87） |  | 12(100.00) | 1(4.76) |  |
| Solidlike enhancing | 2(6.67） | 37(80.43） |  | 0 | 20(95.24) |  |
| Number |  |  | 0.024 |  |  | 0.244 |
| Single | 23(76.67) | 22(47.83) |  | 9(75.00) | 10(47.62) |  |
| Multiple | 7(23.33) | 24(52.17) |  | 3(25.00) | 11(52.38) |  |
| Localization |  |  | 0.017 |  |  | 0.567 |
| Midline | 11(36.67) | 31(67.39) |  | 6(50.00) | 14(66.67) |  |
| Peripheral | 19(63.33) | 15(32.61) |  | 6(50.00) | 7(33.33) |  |
| edema(<1.5) |  |  | 0.804 |  |  | 0.914 |
| Yes | 5(16.67) | 10(21.74) |  | 3(25.00) | 7(33.33) |  |
| No | 25(83.33) | 36(78.26) |  | 9(75.00) | 14(66.67) |  |
| Cystic |  |  | <0.001 |  |  | 0.002 |
| Present | 5(16.67) | 29(63.04) |  | 1(8.33) | 15(71.43) |  |
| Absent | 25(83.33) | 17(36.96) |  | 11(91.67) | 6(28.57) |  |

**Table S4 Radiomics feature of single-sequence and multi-sequence**

|  | Feature Names |
| --- | --- |
| CET1WI | Firstorder_ Skewness_CET1WI |
| (n=7) | GLSZM_ZoneEntropy_CET1WI |
|  | GLRLM_ShortRunHighGrayLevelEmphasis_CET1WI |
|  | Firstorder_RobustMeanAbsoluteDeviation_CET1WI |
|  | GLRLM_ RunEntropy_CET1WI |
|  | GLRLM_ShortRunLowGrayLevelEmphasis_CET1WI |
|  | Firstorder_RootMeanSquared_CET1WI |
| DWI | GLRLM_ShortRunHighGrayLevelEmphasis_DWI |
| (n=4) | GIrlm_LongRunEmphasis_DWI |
|  | GLSZM_ZoneEntropy_DWI |
|  | Shape_ Surface VolumeRatio_DWI |
| FLAIR | Shape_Elongation_FLAIR |
| (n=6) | Firstorder_Range_FLAIR |
|  | Shape_VoxelVolume_FLAIR |
|  | GLRLM_LongRunHighGrayLevelEmphasis_ FLAIR |
|  | Firstorder_Skewness_FLAIR |
|  | GLRLM_ ShortRunLowGrayLevelEmphasis_FLAIR |
| ADC | GLRLM_RunLengthNonUniformity_ADC |
| (n=5) | NGTDM_Busyness_ADC |
|  | GLDM_DependenceEntropy_ADC |
|  | GLRLM_LongRunHighGrayLevelEmphasis_ADC |
|  | Shape_Surface VolumeRatio_ADC |
| CET1WI+FLAIR | Firstorder_Skewness_CET1WI |
| (n=12) | Firstorder _RobustMeanAbsoluteDeviation_CETIWI |
|  | GLRLM_ShortRunHighGrayLevelEmphasis_CET1WI |
|  | Firstorder Range_ FLAIR |
|  | Firstorder_TotalEnergy_FLAIR |
|  | Shape_Elongation_FLAIR |
|  | GLSZM_Zone Variance_CET1WI |
|  | NGTDM_Contrast_CET1WI |
|  | GLSZM_ZonePercentage_CET1WI |
|  | GLRLM_ShortRunLowGrayLevelEmphasis_CET1WI |
|  | GLRLM_RunEntropy_CET1WI |
|  | Firstorder_ RootMeanSquared_CET1WI |
| CE-T1WI+DWI | GLSZM_ZoneEntropy_CETIWI |
| (n=7) | Firstorder Skewness CET1WI |
|  | GLRLM_ShortRunHighGrayLevelEmphasis_CET1WI |
|  | GLRLM Run Variance _DWI |
|  | Firstorder_Kurtosis_CET1WI |
|  | Firstorder_RootMeanSquared_CET1WI |
|  | Shape_Surface VolumeRatio_DWI |
| CE-T1WI+ADC | GLRLM_ShortRunHighGrayLevelEmphasis_CET1WI |
| (n=12) | Firstorder_Skewness_CET1WI |
|  | GLSZM_LowGrayLevelZoneEmphasis_CET1WI |
|  | Firstorder_RobustMeanAbsoluteDeviation_CET1WI |
|  | NGTDM_Busyness_ADC |
|  | GLSZM_LargeArealowGrayLevelEmphasis CETIWI |
|  | GLSZM_ZonePercentage_CET1WI |
|  | NGTDM_Contrast_CET1WI |
|  | GLRLM_RunEntropy_CET1WI |
|  | GLRLM_LongRunLowGrayLevelEmphasis_CET1WI |
|  | GLRLM_ShortRunLowGrayLevelEmphasis_CET1WI |
|  | Firstorder_RootMeanSquared_CET1WI |
| FLAIR+DWI | GLRLM_ShortRunHighGrayLevelEmphasis_DWI |
| (n=6) | NGTDM_Busyness_ADC |
|  | GLSZM_ZoneEntropy_DWI |
|  | GLRLM_LongRunEmphasis_DWI |
|  | Firstorder_Skewness_FLAIR |
|  | Shape_Surface VolumeRatio_DWI |
| FLAIR+ADC | NGTDM_Busyness_ADC |
| (n=7) | GLRLM_RunLengthNonUniformity_ADC |
|  | Shape_Elongation_FLAIR |
|  | Firstorder Range_FLAIR |
|  | Shape_Voxel_Volume_ ADC |
|  | Firstorder_Skewness_FLAIR |
|  | GLRLM_ ShortRunLowGrayLevelEmphasis FLAIR |
| DWI+ADC | GIrlm_ShortRunHighGrayLevelEmphasis_DWI |
| (n=12) | GLRLM_ LongRunEmphasis_DWI |
|  | NGTDM_Busyness_ADC |
|  | GLSZM_ZoneEntropy_DWI |
|  | GLRLM_LongRunHighGrayLevelEmphasis_ADC |
|  | GLSZM_GrayLevelNonUniformity_DWI |
|  | Firstorder_Variance_DWI |
|  | NGTDM_Coarseness_ DWI |
|  | NGTDM Strength DWI |
|  | Shape_Flatness_DWI |
|  | GLCM_Cluster_Shade_DWI |
|  | Shape SurfaceVolumeRatio_DWI |
| CE-T1WI+FLAIR+ADC | GLRLM_ShortRunHighGrayLevelEmphasis_CET1WI |
| (n=18) | Firstorder_TotalEnergy_FLAIR |
|  | GLSZM_Zone Variance_CETIWI |
|  | Firstorder_RobustMeanAbsoluteDeviation_CET1WI |
|  | NGTDM_Busyness_ADC |
|  | Firstorder_Range_FLAIR |
|  | Firstorder_Skewness_CETIWI |
|  | GLSZM_LargeAreaHighGrayLevelEmphasis_ADC |
|  | GLSZM_LowGrayLevelZoneEmphasis_CET1WI |
|  | Shape_Elongation FLAIR - |
|  | Firstorder_ Skewness_FLAIR |
|  | GLRLM_RunLengthNonUniformity_CET1WI |
|  | GLRLM_RunLengthNonUniformityNormalized_ FLAIR |
|  | NGTDM_Contrast_CETIWI |
|  | GLRLM_ShortRunLowGrayLevelEmphasis_CET1W1 |
|  | Firstorder_RootMeanSquared_CET1WI |
|  | GLRLM_RunEntropy_CET1WI |
|  | Shape_LeastAxisLength_FLAIR |
| DWI+ADC+FLAIR | GLRLM_ShortRunHighGrayLevelEmphasis_DWI |
| (n=6) | NGTDM_Busyness_ADC |
|  | GLSZM_ZoneEntropy_DWI |
|  | GLRLM_LongRunEmphasis_DWI |
|  | Firstorder_Skewness_FLAIR |
|  | Shape_Surface VolumeRatio_DWI |
| CE-T1WI+DWI+ADC | GLRLM_ShortRunHighGrayLevelEmphasis_CET1WI |
| (n=21) | GLRLM_ ShortRunHighGrayLevelEmphasis_DWI |
|  | Firstorder_Skewness_CET1WI |
|  | GLRLM_LongRunEmphasis_DWI |
|  | NGTDM_Busyness_ADC |
|  | GLSZM_LowGrayLevelZoneEmphasis_CET1WI |
|  | Firstorder_RobustMeanAbsoluteDeviation_CETIWI |
|  | GLSZM_LargeAreaHighGrayLevelEmphasis_ADC |
|  | GLSZM_LargeAreaLowGrayLevelEmphasis_CET1WI |
|  | GLSZM_Zone Variance_CET1WI |
|  | NGTDM_Contrast_DWI |
|  | GLSZM_ZonePercentage_CET1WI |
|  | NGTDM_Contrast_CET1WI |
|  | Firstorder_Kurtosis_CET1WI |
|  | Shape_Surface VolumeRatio_DWI |
|  | GLRLM_LongRunLowGrayLevelEmphasis_CET1WI |
|  | GLRLM_ShortRunLowGrayLevelEmphasis_CET1WI |
|  | Glem_ClusterShade_DWI |
|  | GLRLM_RunLengthNonUniformity_CET1WI |
|  | Firstorder_RootMeanSquared_CET1WI |
|  | GLRLM_RunEntropy_CET1WI |
| CE-T1WI+FLAIR+DWI | GLRLM_LongRunEmphasis_DWI |
| (n=15) | Firstorder_Skewness_CET1WI |
|  | GLRLM_ ShortRunHighGrayLevelEmphasis_DWI |
|  | Firstorder_TotalEnergy_FLAIR |
|  | GLSZM_ZoneEntropy_CET1WI |
|  | Firstorder_RobustMeanAbsoluteDeviation_CET1WI |
|  | GLRLM_ShortRunHighGrayLevelEmphasis_CET1WI |
|  | Shape_Elongation_FLAIR |
|  | Firstorder_Variance_DWI |
|  | Firstorder _Range_FLAIR |
|  | GLDM_LargeDependenceLowGrayLevelEmphasis_CET1WI |
|  | GLRLM_LongRunLowGrayLevelEmphasis_CET1WI |
|  | NGTDM_Contrast_CET1WI |
|  | Firstorder_Kurtosis_CET1WI |
|  | Firstorder_RootMeanSquared_CET1WI |
| CE-T1WI+FLAIR+DWI+ADC | Firstorder_Skewness_CETTWI |
| (n=13) | GLSZM_Zonentropy_CE11WI |
|  | GLRLM_ShortRunHighGrayLevelEmphasis_CETIWI |
|  | NGTDM_Busyness_ADC |
|  | GLRLM_RunVariance_DWI |
|  | GLRLM_ShortRunHighGrayLevelEmphasis_DWI |
|  | Firstorder_TotalEnergy_FLAIR |
|  | GLRLM_LongRunEmphasis_DWI |
|  | GLDM_LargeDependenceLowGrayLevelEmphasis_CETIWI |
|  | NGTDM_Coarseness_DWI |
|  | Firstorder_Kurtosis_CETIWI |
|  | Firstorder_RootMeanSquared_CETIWI |
|  | Shape_SurfaceVolumeRatio_DWI |

GLRLM=Gray Level Run Length Matrix; GLDM=Gray Level Dependence Matrix；GLSZM= Gray Level Size Zone Matrx; NGTDM= Neighbouring Gray Tone Difference Matrix；GLSZM=Gray Level Size Zone Matrix；GLCM= Gray Level Cooccurence Matrix

**Table S5 Performance of seven models for single-sequence and multi-sequence**

| Model | Training cohort (n=115) | | | | External validation cohort (n=50) | | | |
| --- | --- | --- | --- | --- | --- | --- | --- | --- |
|  | AUC | ACC | Sen | Spe | AUC | ACC | Sen | Spe |
| CE-T1WI |  |  |  |  |  |  |  |  |
| LR | 0.928 | 0.835 | 0.768 | 0.935 | 0.791 | 0.740 | 0.793 | 0.667 |
| SVM | 0.943 | 0.870 | 0.841 | 0.913 | 0.837 | 0.760 | 0.793 | 0.714 |
| RF | 0.953 | 0.887 | 0.884 | 0.891 | 0.823 | 0.740 | 0.724 | 0.762 |
| KNN | 0.865 | 0.722 | 0.609 | 0.891 | 0.796 | 0.660 | 0.552 | 0.810 |
| ExtraTrees | 0.909 | 0.817 | 0.783 | 0.870 | 0.810 | 0.740 | 0.828 | 0.619 |
| LightGBM | 0.923 | 0.870 | 0.855 | 0.891 | 0.775 | 0.740 | 0.828 | 0.619 |
| MLP | 0.908 | 0.809 | 0.797 | 0.826 | 0.816 | 0.760 | 0.897 | 0.571 |
| DWI |  |  |  |  |  |  |  |  |
| LR | 0.830 | 0.757 | 0.696 | 0.848 | 0.775 | 0.720 | 0.586 | 0.905 |
| SVM | 0.871 | 0.809 | 0.739 | 0.913 | 0.757 | 0.780 | 0.690 | 0.905 |
| RF | 0.889 | 0.800 | 0.696 | 0.957 | 0.732 | 0.680 | 0.517 | 0.905 |
| KNN | 0.865 | 0.643 | 0.406 | 1.000 | 0.672 | 0.540 | 0.276 | 0.905 |
| ExtraTrees | 0.847 | 0.748 | 0.623 | 0.935 | 0.646 | 0.600 | 0.379 | 0.905 |
| LightGBM | 0.874 | 0.817 | 0.652 | 0.913 | 0.732 | 0.660 | 0.552 | 0.810 |
| MLP | 0.832 | 0.748 | 0.623 | 0.935 | 0.801 | 0.780 | 0.690 | 0.905 |
| ADC |  |  |  |  |  |  |  |  |
| LR | 0.741 | 0.713 | 0.783 | 0.609 | 0.678 | 0.680 | 0.828 | 0.476 |
| SVM | 0.796 | 0.730 | 0.841 | 0.674 | 0.657 | 0.640 | 0.724 | 0.524 |
| RF | 0.903 | 0.800 | 0.696 | 0.957 | 0.625 | 0.600 | 0.517 | 0.714 |
| KNN | 0.809 | 0.652 | 0.507 | 0.870 | 0.583 | 0.500 | 0.276 | 0.810 |
| ExtraTrees | 0.807 | 0.722 | 0.696 | 0.761 | 0.615 | 0.620 | 0.724 | 0.476 |
| LightGBM | 0.832 | 0.774 | 0.681 | 0.804 | 0.617 | 0.640 | 0.862 | 0.333 |
| MLP | 0.737 | 0.704 | 0.739 | 0.652 | 0.700 | 0.658 | 0.897 | 0.429 |
| FLAIR |  |  |  |  |  |  |  |  |
| LR | 0.770 | 0.670 | 0.522 | 0.891 | 0.750 | 0.740 | 0.655 | 0.857 |
| SVM | 0.831 | 0.730 | 0.609 | 0.913 | 0.687 | 0.680 | 0.793 | 0.524 |
| RF | 0.911 | 0.817 | 0.768 | 0.891 | 0.787 | 0.780 | 0.759 | 0.810 |
| KNN | 0.845 | 0.565 | 0.275 | 0.975 | 0.713 | 0.660 | 0.724 | 0.571 |
| ExtraTrees | 0.860 | 0.739 | 0.638 | 0.913 | 0.755 | 0.660 | 0.517 | 0.857 |
| LightGBM | 0.863 | 0.739 | 0.594 | 0.957 | 0.722 | 0.720 | 0.621 | 0.857 |
| MLP | 0.762 | 0.687 | 0.594 | 0.826 | 0.757 | 0.720 | 0.586 | 0.905 |
| CE-T1WI+FLAIR |  |  |  |  |  |  |  |  |
| LR | 0.963 | 0.896 | 0.913 | 0.870 | 0.824 | 0.740 | 0.621 | 0.905 |
| SVM | 0.971 | 0.896 | 0.841 | 0.978 | 0.833 | 0.780 | 0.931 | 0.571 |
| RF | 0.986 | 0.939 | 0.928 | 0.957 | 0.813 | 0.760 | 0.931 | 0.524 |
| KNN | 0.904 | 0.757 | 0.652 | 0.913 | 0.800 | 0.620 | 0.483 | 0.810 |
| ExtraTrees | 0.923 | 0.917 | 0.739 | 0.935 | 0.815 | 0.760 | 0.828 | 0.667 |
| LightGBM | 0.937 | 0.869 | 0.739 | 0.957 | 0.777 | 0.740 | 0.862 | 0.571 |
| MLP | 0.915 | 0.835 | 0.855 | 0.804 | 0.844 | 0.780 | 0.897 | 0.619 |
| CE-T1WI+DWI |  |  |  |  |  |  |  |  |
| LR | 0.902 | 0.835 | 0.812 | 0.870 | 0.816 | 0.760 | 0.724 | 0.810 |
| SVM | 0.942 | 0.861 | 0.797 | 0.957 | 0.826 | 0.760 | 0.793 | 0.714 |
| RF | 0.973 | 0.922 | 0.913 | 0.935 | 0.816 | 0.760 | 0.690 | 0.857 |
| KNN | 0.911 | 0.800 | 0.725 | 0.913 | 0.816 | 0.660 | 0.517 | 0.857 |
| ExtraTrees | 0.898 | 0.809 | 0.783 | 0.848 | 0.778 | 0.740 | 0.655 | 0.857 |
| LightGBM | 0.944 | 0.878 | 0.913 | 0.826 | 0.795 | 0.740 | 0.724 | 0.762 |
| MLP | 0.903 | 0.817 | 0.812 | 0.826 | 0.828 | 0.760 | 0.655 | 0.905 |
| CE-T1WI+ADC |  |  |  |  |  |  |  |  |
| LR | 0.959 | 0.896 | 0.870 | 0.935 | 0.772 | 0.720 | 0.690 | 0.762 |
| SVM | 0.967 | 0.896 | 0.841 | 0.978 | 0.823 | 0.760 | 0.759 | 0.762 |
| RF | 0.960 | 0.896 | 0.884 | 0.935 | 0.701 | 0.680 | 0.759 | 0.571 |
| KNN | 0.910 | 0.774 | 0.667 | 0.935 | 0.771 | 0.680 | 0.586 | 0.810 |
| ExtraTrees | 0.924 | 0.852 | 0.826 | 0.891 | 0.791 | 0.720 | 0.759 | 0.667 |
| LightGBM | 0.945 | 0.878 | 0.870 | 0.891 | 0.719 | 0.680 | 0.586 | 0.810 |
| MLP | 0.935 | 0.870 | 0.913 | 0.804 | 0.826 | 0.780 | 0.931 | 0.571 |
| FLAIR+DWI |  |  |  |  |  |  |  |  |
| LR | 0.877 | 0.809 | 0.725 | 0.935 | 0.828 | 0.760 | 0.621 | 0.952 |
| SVM | 0.900 | 0.852 | 0.797 | 0.935 | 0.831 | 0.820 | 0.828 | 0.810 |
| RF | 0.951 | 0.887 | 0.884 | 0.891 | 0.678 | 0.660 | 0.517 | 0.857 |
| KNN | 0.857 | 0.748 | 0.623 | 0.935 | 0.726 | 0.600 | 0.345 | 0.952 |
| ExtraTrees | 0.888 | 0.812 | 0.754 | 0.913 | 0.792 | 0.740 | 0.690 | 0.810 |
| LightGBM | 0.901 | 0.817 | 0.768 | 0.891 | 0.792 | 0.740 | 0.724 | 0.762 |
| MLP | 0.865 | 0.817 | 0.841 | 0.783 | 0.857 | 0.800 | 0.724 | 0.905 |
| FLAIR+ADC |  |  |  |  |  |  |  |  |
| LR | 0.806 | 0.739 | 0.725 | 0.761 | 0.736 | 0.740 | 0.690 | 0.810 |
| SVM | 0.876 | 0.748 | 0.594 | 0.978 | 0.727 | 0.700 | 0.655 | 0.762 |
| RF | 0.934 | 0.887 | 0.942 | 0.804 | 0.705 | 0.680 | 0.552 | 0.857 |
| KNN | 0.884 | 0.704 | 0.522 | 0.978 | 0.760 | 0.560 | 0.241 | 1.000 |
| ExtraTrees | 0.845 | 0.800 | 0.826 | 0.761 | 0.690 | 0.640 | 0.414 | 0.952 |
| LightGBM | 0.889 | 0.817 | 0.783 | 0.870 | 0.790 | 0.720 | 0.724 | 0.714 |
| MLP | 0.806 | 0.713 | 0.623 | 0.848 | 0.785 | 0.780 | 0.690 | 0.905 |
| DWI+ADC |  |  |  |  |  |  |  |  |
| LR | 0.862 | 0.809 | 0.913 | 0.652 | 0.765 | 0.680 | 0.517 | 0.905 |
| SVM | 0.890 | 0.817 | 0.710 | 0.978 | 0.773 | 0.780 | 0.690 | 0.905 |
| RF | 0.922 | 0.817 | 0.768 | 0.891 | 0.730 | 0.640 | 0.483 | 0.857 |
| KNN | 0.871 | 0.626 | 0.377 | 1.000 | 0.763 | 0.620 | 0.414 | 0.905 |
| ExtraTrees | 0.849 | 0.774 | 0.754 | 0.804 | 0.768 | 0.700 | 0.586 | 0.857 |
| LightGBM | 0.893 | 0.809 | 0.826 | 0.783 | 0.741 | 0.700 | 0.759 | 0.619 |
| MLP | 0.859 | 0.765 | 0.710 | 0.848 | 0.793 | 0.760 | 0.690 | 0.857 |
| CE-T1WI+FLAIR+ADC |  |  |  |  |  |  |  |  |
| LR | 0.989 | 0.848 | 0.928 | 0.978 | 0.816 | 0.760 | 0.724 | 0.810 |
| SVM | 0.979 | 0.913 | 0.899 | 0.935 | 0.844 | 0.780 | 0.724 | 0.857 |
| RF | 0.980 | 0.922 | 0.884 | 0.978 | 0.750 | 0.700 | 0.690 | 0.714 |
| KNN | 0.914 | 0.748 | 0.623 | 0.935 | 0.767 | 0.600 | 0.345 | 0.952 |
| ExtraTrees | 0.923 | 0.889 | 0.710 | 0.957 | 0.826 | 0.720 | 0.586 | 0.905 |
| LightGBM | 0.955 | 0.904 | 0.884 | 0.935 | 0.746 | 0.700 | 0.655 | 0.762 |
| MLP | 0.955 | 0.887 | 0.870 | 0.913 | 0.819 | 0.720 | 0.621 | 0.857 |
| CE-T1WI+FLAIR+DWI |  |  |  |  |  |  |  |  |
| LR | 0.952 | 0.870 | 0.855 | 0.891 | 0.829 | 0.780 | 0.966 | 0.524 |
| SVM | 0.975 | 0.904 | 0.853 | 0.978 | 0.871 | 0.837 | 0.821 | 0.857 |
| RF | 0.948 | 0.913 | 0.870 | 0.978 | 0.851 | 0.800 | 0.862 | 0.714 |
| KNN | 0.915 | 0.791 | 0.696 | 0.935 | 0.848 | 0.760 | 0.759 | 0.762 |
| ExtraTrees | 0.922 | 0.861 | 0.928 | 0.761 | 0.804 | 0.720 | 0.724 | 0.714 |
| LightGBM | 0.950 | 0.887 | 0.913 | 0.848 | 0.805 | 0.700 | 0.517 | 0.952 |
| MLP | 0.931 | 0.826 | 0.768 | 0.913 | 0.859 | 0.820 | 0.897 | 0.714 |
| CE-T1WI+DWI+ADC |  |  |  |  |  |  |  |  |
| LR | 0.974 | 0.904 | 0.870 | 0.957 | 0.816 | 0.740 | 0.621 | 0.905 |
| SVM | 0.970 | 0.822 | 0.884 | 0.978 | 0.852 | 0.820 | 0.793 | 0.857 |
| RF | 0.975 | 0.813 | 0.884 | 0.957 | 0.814 | 0.760 | 0.759 | 0.762 |
| KNN | 0.923 | 0.800 | 0.739 | 0.891 | 0.856 | 0.740 | 0.586 | 0.952 |
| ExtraTrees | 0.946 | 0.894 | 0.841 | 0.913 | 0.784 | 0.760 | 0.724 | 0.810 |
| LightGBM | 0.946 | 0.894 | 0.855 | 0.935 | 0.756 | 0.720 | 0.759 | 0.667 |
| MLP | 0.942 | 0.878 | 0.856 | 0.913 | 0.824 | 0.780 | 0.793 | 0.762 |
| FLAIR+ADC+DWI |  |  |  |  |  |  |  |  |
| LR | 0.861 | 0.809 | 0.841 | 0.761 | 0.750 | 0.660 | 0.483 | 0.905 |
| SVM | 0.917 | 0.791 | 0.667 | 0.978 | 0.782 | 0.720 | 0.690 | 0.762 |
| RF | 0.923 | 0.861 | 0.899 | 0.804 | 0.726 | 0.700 | 0.586 | 0.857 |
| KNN | 0.872 | 0.722 | 0.580 | 0.935 | 0.731 | 0.560 | 0.345 | 0.857 |
| ExtraTrees | 0.863 | 0.774 | 0.725 | 0.848 | 0.613 | 0.620 | 0.414 | 0.905 |
| LightGBM | 0.899 | 0.774 | 0.667 | 0.935 | 0.741 | 0.680 | 0.759 | 0.571 |
| MLP | 0.870 | 0.800 | 0.826 | 0.761 | 0.782 | 0.720 | 0.690 | 0.762 |
| CET1WI+FLAIR+DWI+ADC |  |  |  |  |  |  |  |  |
| LR | 0.928 | 0.870 | 0.884 | 0.848 | 0.829 | 0.740 | 0.724 | 0.762 |
| SVM | 0.960 | 0.869 | 0.855 | 0.957 | 0.844 | 0.780 | 0.690 | 0.905 |
| RF | 0.970 | 0.939 | 0.942 | 0.935 | 0.860 | 0.820 | 0.759 | 0.905 |
| KNN | 0.939 | 0.826 | 0.754 | 0.935 | 0.858 | 0.720 | 0.586 | 0.905 |
| ExtraTrees | 0.920 | 0.809 | 0.710 | 0.957 | 0.825 | 0.740 | 0.586 | 0.952 |
| LightGBM | 0.954 | 0.861 | 0.783 | 0.978 | 0.801 | 0.760 | 0.724 | 0.810 |
| MLP | 0.931 | 0.843 | 0.797 | 0.913 | 0.831 | 0.760 | 0.621 | 0.952 |

**Table S6 Radiomics features of RF、LightGBM and ET**

| Model | Feature |  |
| --- | --- | --- |
| RF (n=15) | Firstorder_Skewness_CET1WI |  |
|  | GLSZM_ZoneEntropy_CET1WI |  |
|  | GLRLM_ShortRunHighGrayLevelEmphasis_DWI |  |
|  | GLRLM_ShortRunHighGrayLevelEmphasis_CET1WI |  |
|  | Firstorder_RootMeanSquared_CET1WI |  |
|  | Firstorder_TotalEnergy_FLAIR |  |
|  | Firstorder_Range_FLAIR |  |
|  | Firstorder_Variance_DWI |  |
|  | Firstorder_Kurtosis_CET1WI |  |
|  | Shape_Elongation_FLAIR |  |
|  | GLRLM_LongRunLowGrayLevelEmphasis_CET1WI |  |
|  | NGTDM_Contrast_CET1WI |  |
|  | Firstorder_RobustMeanAbsoluteDeviation_CET1WI |  |
|  | GLRLM_LongRunEmphasis_DWI |  |
|  | GLDM_LargeDependenceLowGrayLevelEmphasis_CET1WI |  |
| LightGBM(n=13) | Firstorder_RootMeanSquared_CET1WI |  |
|  | Firstorder_Skewness_CET1WI |  |
|  | GLRLM_ShortRunHighGrayLevelEmphasis_CET1WI |  |
|  | GLSZM_ZoneEntropy_CET1WI |  |
|  | GLRLM_ShortRunHighGrayLevelEmphasis_DWI |  |
|  | Firstorder_TotalEnergy_FLAIR |  |
|  | Firstorder_RobustMeanAbsoluteDeviation_CET1WI |  |
|  | Firstorder_Range_FLAIR |  |
|  | Firstorder_Kurtosis_CET1WI |  |
|  | GLDM_LargeDependenceLowGrayLevelEmphasis_CET1WI |  |
|  | Firstorder_Variance_DWI |  |
|  | GLRLM_LongRunEmphasis_DWI |  |
| ET(n=15) | GLRLM_ShortRunHighGrayLevelEmphasis_CET1WI |  |
|  | GLRLM_ShortRunHighGrayLevelEmphasis_DWI |  |
|  | GLDM_LargeDependenceLowGrayLevelEmphasis_CET1WI |  |
|  | GLSZM_ZoneEntropy_CET1WI |  |
|  | Firstorder_RootMeanSquared_CET1WI |  |
|  | Firstorder_Range_FLAIR |  |
|  | GLRLM_LongRunEmphasis_DWI |  |
|  | Shape_Elongation_FLAIR |  |
|  | Firstorder_RobustMeanAbsoluteDeviation_CET1WI |  |
|  | Firstorder_Kurtosis_CET1WI |  |
|  | GLRLM_LongRunLowGrayLevelEmphasis_CET1WI |  |
|  | Firstorder_Skewness_CET1WI |  |
|  | NGTDM_Contrast_CET1WI |  |
|  | Firstorder_TotalEnergy_FLAIR |  |

**
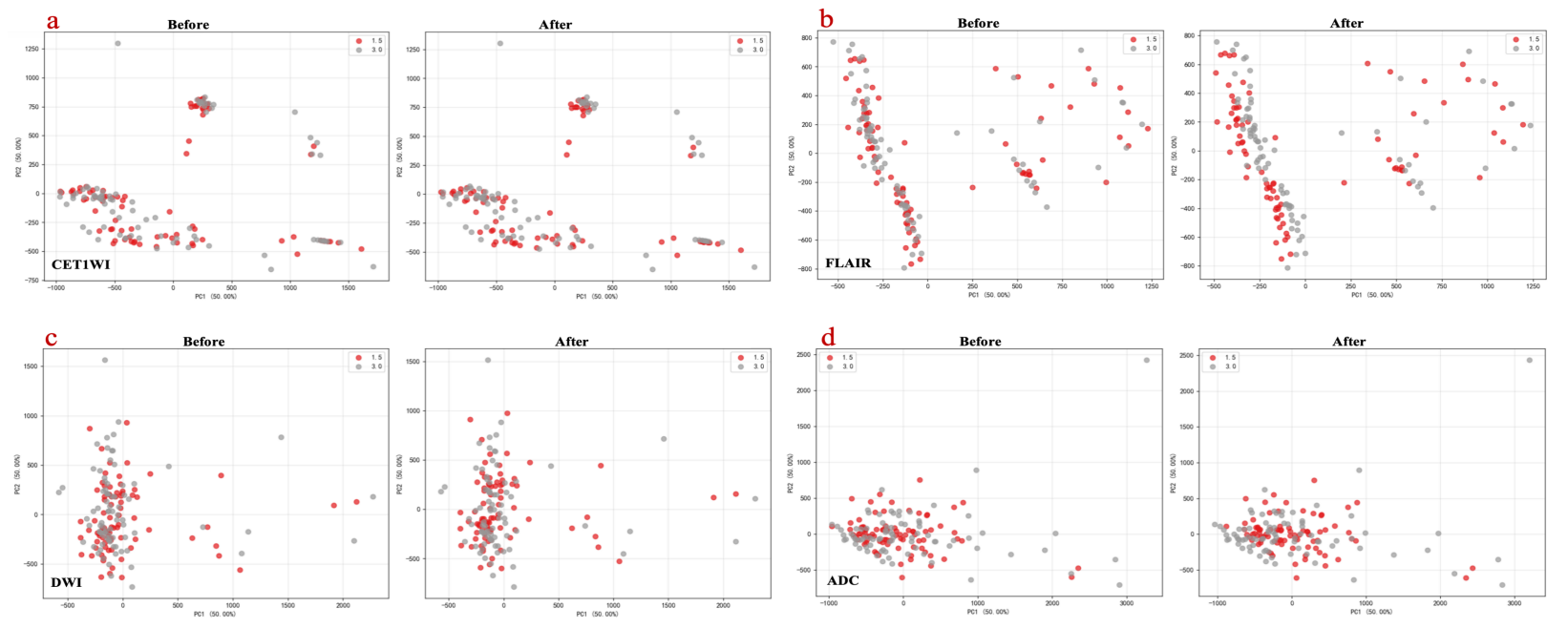
Figure S1 PCA before and after calibration of CET1WI, FLAIR, DWI and ADC**

**Supplementary Information 1**

**ADG stratified materials of training and external validation cohort**

**Training cohort**

IDH-mutant astrocytoma（n=30 ，age=45.03±15.08， female=14， male=16）

oligodendroglioma（n=9 ，age=48.67±10.67， female=5， male=4）

IDH-wildtype glioblastoma（n=30，age=51.67±17.68， female=11， male=19）

**External validation cohort**

IDH-mutant astrocytoma（n=13 ，age=37.23±15.47， female=7， male=6）

oligodendroglioma（n=4 ，age=48.25±17.23， female=1， male=3）

IDH-wildtype glioblastoma（n=12，age=55.17±11.83， female=5， male=7）

**Supplementary Information 2**

**The hyperparameters search space of each machine learning model**

**'LR'** = LogisticRegression (penalty=' none' , max_iter=[50, 100, 200])

**' SVM' =** SVC (probability=[True, False], max_iter=[50, 100, 200], kernel=['linear', 'rbf'])

**' RandomForest'** = RandomForestClassifier (n_estimators=[5,10], max_depth=(3, 5, 10), min_samples_split=[4, 5], random_state=0)

**' LightGBM' =** LGBMClassifier (n_estimators=[2, 3, 5], max_depth=[1, 3, 5], min_child_weight=[0.5, 1])

**' ExtraTrees' =** ExtraTreesClassifier (n_estimators=[50, 60, 100], max_depth=[3, 5, 7], min_samples_split=[1, 2, 4], random_state=0)

**' KNN' =** KNNClassifier (n_neighbors=[3, 5, 7])

**' MLP' =** MLPClassifier (hidden_layer_sizes= (61, 128, 64, 32), max_iter=[100, 200, 300]. solver='sgd', random_state=0)
